# Connecticut Valley Dental Society

**Published:** 1887-09

**Authors:** Geo. A. Maxfield

**Affiliations:** Secretary


### CONNECTICUT VALLEY DENTAL SOCIETY.

SEMI-ANNUAL MEETING, HELD AT MONTREAL, P. Q., JULY 19 TO 21, INCLUSIVE.

REPORTED BY GEO. A. MAXFIELD, D. D. S., SECRETARY.

The meeting was called to order at 10.30 A. M., President Stebbins in the chair.

C. F. Trestler, M. D., L. D. S., President of the Board of Trustees and Examiners of the Dental Association of the Province of Quebec, welcomed the society to Montreal as follows :

*Mr. President, ladies and members of the Connecticut Valley Dental Society :*

As President of the Board of Examiners of the Dental Association of the Province of Quebec, it gives me the very greatest pleasure to extend to you our humble welcome. While appreciating how much we are both indebted for a great deal of the theory and science of dentistry to the investigation of British, French and German savants, we know that to the zealous and persevering practical work of our American cousins, dentistry owes the position it occupies among the learned professions of the day. Your conventions have become dental missionary conquests, where doubt is dissipated and the light of truth is revealed. We feel honored, as we feel sure we shall be profited, by your presence in Montreal, and we trust that you may all—especially the ladies—carry home with you nothing but pleasant recollections of your first visit to the metropolis of our Dominion. Let me once more, Mr. President, ladies and gentlemen, reiterate the gratification we enjoy in welcoming you individually and collectively to Montreal.

Dr. Stebbins, President of the Society, responded as follows :

Mr. President, allow me to return you and your Association sincere thanks on behalf of the Connecticut Valley Dental Society, for this cordial welcome to your beautiful and renowned city, and especially for your hearty greetings. With you, we ascribe all honor due the learned of our profession on the other side of the water. I suppose it is universally conceded, that in America dentistry has attained a higher standard and made more marvelous progress than in any other country. I believe that New York City lays claim to being the birth-place of many things that have contributed to make our profession what it is. This society comes to sit with you in fraternal council, and discuss those topics that are of mutual interest. We have not dispelled all darkness, nor have we apprehended all truth concerning our calling, but having apprehended some, we press forward. We have anticipated much of good and pleasure in coming to Montreal, and the manner in which we are received reassures us those anticipations will be more than realized.

We considered it appropriate to bring our queens with us to visit the land which has for fifty years been ruled so graciously and beneficently by that Christian Queen of queens—Queen Victoria. When Dr. Lovejoy invited the society to hold this meeting here, in his eloquent speech he said we must bear in mind we were to visit a foreign country. I observe nothing foreign about this occasion. It seems to me we are one people. Some young ladies (in the States) of vivacious natures are impelled to make alliances and engage in the affairs of life contrary to parental wishes, and even in violation of parental injunctions, but when success is attained and fortune smiles on the venture, parental love triumphs over displeasure, and the children of the once deemed wayward girls are welcome at the family domicile.

One hundred and eleven years ago this month, thirteen colonial daughters entered into an alliance with the American Eagle, and

"set up house-keeping" on their own account. I am informed that the parent Government was not well pleased at first, but finally withdrew its objections to the union and entered into friendly relations. We are descendants of those daughters, and come to visit the parental domain and enjoy the rich things prepared for us. In looking over the bill of fare (programme), I observe not only that various dishes are given, but the cooks are named who have prepared them for us. Such a display of names and articles assures us we are to be entertained and fed. It is with pleasure I extend you, collectively and individually, an earnest and cordial invitation to attend the meetings of our society whenever it may be your pleasure to do so, and we will endeavor to give you a hearty reception.

[The abstract and discussion of Dr. W. Geo. Beers' paper on "Alveolar Abscess" is left till the next number, that the discussion of Dr. Black's paper may accompany its publication.—EDITOR.]

WEDNESDAY MORNING SESSION.

Meeting called to order at nine o'clock.

Dr. G. V. Black read a paper on "Recent Theories of the Formation of Pus, Considered with Reference to Dental Operations." (See page 462.)

DISCUSSION.

*Dr. Barrett*—Dr. Black has answered the question that I have often propounded to myself: What is pus ? For many years I have been considering this matter, and to-day I get a definite and precise answer. A few years ago I asked Dr. Atkinson that question, and received in return six or eight pages of written matter, but he did not answer the question. I again wrote, and asked him, "what is pus ?" and he once more replied with six or eight pages. Again I wrote, and asked, *what is pus?* and he finally answered, "Pus is dead blood," and this was so indefinite that I closed the catechism. Now I hope we shall have a discussion that will not lead us away out into the woods, a thousand miles from anywhere.

*Dr. Alexander*—I would like to know which of the tissues contains or furnishes the most nutritive elements in supporting alveolar abscess.

*Dr. Black*—The connective tissue.

*Dr. Cook*—What length of time will these microbes live in a healthy tissue ?

*Dr. Black*—If a healthy man's blood is contaminated they will disappear in a few days.

*Dr. Atkinson*—A few hours.

*Dr. Black*—Unless they are of a kind that thrive in the blood.

*Prof. Mayr.*—Dr. Black made an axiomatic statement, that chemical substances would not change of their own account. While a plausible statement, I do not think that it is yet sufficiently proved. As an example, I would mention cyanate of ammonia. You may keep it after crystallization in whatever way you please, it will change in the course of time into the entirely different carburet or urea.

*Dr. Black*—This substance is probably not at any time an actual compound. The elements that have been presented to each other in their passage through certain processes for obtaining an equilibrium form cyanate of ammonia, but the process does not stop at that point, but goes on, until the affinities are fully satisfied. When these chemical processes once stop, they are at a final end.

*Dr. Atkinson*—Dr. Black says: "Pus corpuscles are amœboidal bodies." They are not amœboid until they die, and they are not pus corpuscles until they are deprived of the tissue-building power.

*Dr. Stevens*—Before the speaker goes on further I would ask, what is pus?

*Dr. Atkinson*—I said pus was dead blood, and the sum of what Dr. Black said made it so, too. I would like to see how he distinguishes between live and dead matter. If he sees an amœba that is asleep, he says it is death.

*Prof. Mayr*—To define pus in a few words is very difficult. The definition of Dr. Atkinson, as dead blood, might be misleading. We may take blood and, under proper antiseptic methods, may allow it to die. Blood taken from the body is not dead yet, by any means; it keeps alive for a long time, for days, but it finally ceases to show the characteristic properties of blood as it is in the body, and we may very properly call it dead. Now pus is very different from that. It is not only dead blood, but it is entirely changed chemically. To define pus completely would be like defining a chemical laboratory. I can say it is a shop where glasses, bottles and chemicals are, but that is a drug store too; and pus is the product of such a laboratory, and the whole chemical laboratory itself, where the chemists are microbes and the products are ptomaines.

Subject passed.

